# New Insights into the Fouling of a Membrane during the Ultrafiltration of Complex Organic–Inorganic Feed Water

**DOI:** 10.3390/membranes13030334

**Published:** 2023-03-14

**Authors:** Vedrana Prorok, Dejan Movrin, Nataša Lukić, Svetlana Popović

**Affiliations:** 1 Faculty of Technology Novi Sad, University of Novi Sad, Bulevar Cara Lazara 1, 21000 Novi Sad, Serbia; 2 Faculty of Technical Sciences Novi Sad, University of Novi Sad, Trg Dositeja Obradovića 6, 21000 Novi Sad, Serbia

**Keywords:** membrane fouling resistance, wastewater, humic acid, colloidal silica, ultrafiltration, fouling

## Abstract

This paper presents an analysis of the fouling of a ceramic membrane by a mixture containing high concentrations of humic acid and colloidal silica during cross-flow ultrafiltration under various operating conditions. Two types of feed water were tested: feed water containing humic acid and feed water containing a mixture of humic acid and colloidal silica. The colloidal silica exacerbated the fouling, yielding lower fluxes (109–394 L m^−2^ h^−1^) compared to the humic acid feed water (205–850 L m^−2^ h^−1^), while the retentions were higher except for the highest cross-flow rate. For the humic acid feed water, the irreversible resistance prevails under the cross-flow rate of 5 L min^−1^. During the filtration of an organic–inorganic mixture, the reversible resistance due to the formation of a colloidal cake layer prevails under all operating conditions with an exception. The exception is the filtration of the organic–inorganic mixture of a 50 mg L^−1^ humic acid concentration which resulted in a lower flux than the one of a 150 mg L^−1^ humic acid concentration under 150 kPa and a cross-flow rate of 5 L min^−1^. Here, the irreversible fouling is unexpectedly overcome. This is unusual and occurs due to the low agglomeration at low concentrations of humic acid under a high cross-flow rate. Under lower transmembrane pressure and a moderate cross-flow rate, fouling can be mitigated, and relatively high fluxes are yielded with high retentions even in the presence of nanoparticles. In this way, colloidal silica influences the minimization of membrane fouling by organic humic acid contributing to the control of in-pore organic fouling.

## 1. Introduction

It is well-known that membranes are broadly used in water and wastewater treatment despite the occurrence of membrane fouling, which can reduce the effectiveness of the filtration process. Depending on the type of fouling, it can be controlled in cross-flow filtration by adjusting the operating conditions and by choosing the appropriate membrane type. However, in treated feed waters, various constituents can naturally appear, and their interaction can even exacerbate the fouling of a membrane. Several processes lead to the fouling of membranes such as the adsorption of particles from a solution, the deposition of particles, and gel formation [1,2]. Numerous forms of natural organic and inorganic matter in wastewater cause fouling of the membrane. One of the organic foulants of the membrane is humic acid (HA) present in soil and water, while silica particles (SiO_2_), widely used nanoparticles in many products, can appear as inorganic foulants [3,4,5,6,7,8]. Humic substances can reside in the soil for a very long time due to the interactions with clay minerals and have an impact on the ecosystem, including carbon cycling, nutrient intake, transport, and foulant transfer [9]. Additionally, aggregation, deposition, and transformation processes are naturally occurring, so engineered nanoparticles can be significantly impacted by the adsorption of organic matter [10]. It was discovered, for example, that the presence of Ca^2+^ ions can cause the agglomeration of humic acid as a form of natural organic matter present in wastewater [6]. Hence, it is important to uncover the mechanisms of fouling by humic acid and colloidal silica. Most of the fouling studies have focused on the individual organic or inorganic fouling of a membrane, while mutual fouling effects in membrane filtration remain relatively unidentified. Additionally, many studies have been oriented to improve the characteristics of polymer membranes by adding different fillers for the better removal of humic acid.

Taheri et al. [11] investigated the combined fouling of an ultrafiltration polymeric membrane with humic acid and colloidal silica during dead-end ultrafiltration (UF) at a constant pressure of 210 kPa. They found that humic acid and colloidal silica react differently to intermittent relaxation because of differences in particle size, compressibility, and metastability. Further, they found that frequent relaxation is more effective since it prevents the build-up of a thick cake layer that can lead to internal pore fouling with low compressibility HA. Frequent relaxation is important to minimize the increase in transmembrane pressure to avoid the onset of a transition to a firmer cake layer. Li and Elimelech [12] also studied the combined fouling of a thin-film composite membrane, during the dead-end nanofiltration of Suwannee River humic acid and silica colloids. Qin et al. [13] investigated the synergistic effect of humic acid, BSA, alginate, and colloidal silica on the fouling of a polymeric microporous membrane during membrane distillation. The studied concentrations of humic acid and colloidal silica were lower than 50 and 100 mg L^−1^, respectively.

In addition, the research to date has been based on the investigation of a mixture of the effect of HA and colloidal SiO_2_ mostly on polymer membranes during dead-end filtration. To our knowledge, there is still no research on the effect of this mixture on ceramic membranes by cross-flow UF. Lee et al. [14] described the critical differences between polymeric and ceramic membranes. Ceramic membranes have significantly less irreversible fouling than polymeric ones. The major reason for this is the lower interaction of the foulant with ceramic membranes than with polymeric membranes, due to the more hydrophilic nature of ceramic membranes. Polymer membranes are more economically acceptable, and due to the economy and the possibility of a higher ability to modify their surface chemistry, many studies have focused on improving the characteristics of polymer membranes by impregnating different fillers for the better removal of humic acid [15,16]. However, ceramic membranes have longer lifespans compared to polymeric membranes. Made of inorganic materials, ceramic membranes have a much higher chemical resistivity, enabling much more aggressive cleaning approaches without the risk of damaging the integrity of the membrane.

This paper presents the investigation of the cross-flow ultrafiltration of a complex organic–inorganic mixture on a ceramic membrane. The aim was to analyze fouling by feed water containing humic acid and colloidal silica in high concentrations. Also, the influence of operating conditions, such as transmembrane pressure and cross-flow rate, was studied. New insights were gained into the fouling and retention of HA due to the presence of colloidal silica and its specific interaction with humic acid under a certain combination of operating conditions and concentrations of HA. 

## 2. Materials and Methods

### 2.1. Filtration Experiments

**Figure 1:**
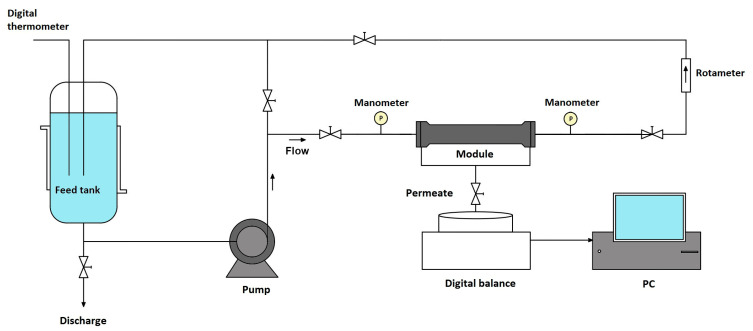
Schematic illustration of the experimental setup for cross-flow filtration.

All the experiments were carried out using the microfiltration/ultrafiltration experimental setup made of stainless steel (Figure 1). The transmembrane pressure (TMP) and cross-flow rate (Q) were regulated using the bypass valve and the main flow valve and monitored by digital manometers and a rotameter. The temperature was monitored by a digital thermometer. The permeate was gathered in the container placed on the digital balance (EW 1500-2 M, KERN, Balingen, DE Germany) and continuously weighed; the obtained data were transferred to a personal computer. 

The single-channel ceramic membrane with a molecular weight cut-off size (MWCO) of 100 kDa, made of a ZrO_2_ filtering layer on an α-alumina support (Atech, Aachen, DE) was used. The ceramic membrane had a length of 250 mm, 6 mm ID (internal diameter), and 10 mm OD (outside diameter). The active membrane surface was 4.71 × 10^−3^ m^2^. The membrane was characterized by pH stability in the range of 0–14 and suitability for steam sterilization at 121 °C. The resistance of the clean membrane (Rm) amounts to 5.39 × 10^11^ m^−1^, received from the water flux measurement at various TMPs. 

Two types of feed water were filtered: organic feed water containing humic acid (Sigma-Aldrich, Saint Louis, MO, USA) and an organic–inorganic feed containing humic acid and colloidal silica (Centrohem, Stara Pazova, Serbia). Model systems of feed waters were prepared by stirring humic acid and colloidal silica in distilled water to obtain 7 L of feed. To ensure proper dissolution, 1 L of distilled water was mixed on a magnetic stirrer (AMTAST Basic, Qingdao, China) for 10 min. The remaining 6 L of distilled water was then added and mixed using an overhead stirrer (VELP scientific, Usmate Velate, IT) for 15 min before being poured into the tank. The studied concentrations of humic acid (HA) were 50, 100, and 150 mg L^−1^, and for colloidal silica, the concentrations were 500 and 1000 mg L^−1^. The experiments were performed under various transmembrane pressures (TMP) (50, 100, and 150 kPa) and cross-flow rates (Q) (1, 3, and 5 L min^−1^). The temperature was kept constant at 25 ± 0.5 °C during all experiments. The pH values in the feed and permeate samples were measured (HACH, Loveland, CO, USA). The pH was 6.6 ± 0.5 during all experiments. The experiments were done in triplicate and the average values were used for calculation. 

The retention of the membrane for humic acid was determined based on the difference in the concentrations of HA in the feed water and permeate. The sampling of the permeate was carried out at certain time intervals up to 120 min (10, 20, 30, 45, 60, 90, and 120 min) to measure the concentration of humic acid. To accurately determine the concentrations of humic acid in the samples, a calibration with standard solutions was performed. A series of standard solutions with humic acid were prepared in distilled water. Then, the absorbance of each standard solution, which was placed in a 4 mL quartz cuvette, was measured at a wavelength of 254 nm, using a UV-visible spectrophotometer (T80 UV/VIS, Hric Group International, London, UK). After measuring the absorbance values of the standard solutions, a graph was plotted to illustrate the relationship between the concentrations of the standard solutions and their corresponding absorbance values. The obtained standard calibration curve (y = 86.53 and R^2^ = 0.996) was used to determine the concentrations of humic acid in the samples. Also, microscopic images of the organic–inorganic feed water were produced on a light microscope (Olympus BX41, Tokyo, Japen) under a 10× *g* magnification.

After the filtration part of the experiment, the membrane was rinsed with distilled water for 15 min under the same conditions that the filtration was conducted under to remove surface fouling. Then, the fresh distilled water was fed, and the flux of water (Jpire) was measured under the same conditions that the filtration was conducted under. This flux served to determine the irreversible and reversible resistances of the membrane. After each experiment, the membrane was cleaned by applying the three-step base-acid-base procedure. 

### 2.2. Calculations

The flux of the permeate (*Jp*) was calculated by the following equation:

(1) $$Jp=\frac{V}{t*A_{ac}}\;\left[L\;m^{-2}\;h^{-1}\right]$$

where *V* isthe volume of permeate (L); tis the time for which the volume was collected (h); *A_ac_* isthe active surface area of the membrane (m^2^) [17].

Retention (*R*) was calculated by the following equation:

(2) $$R=\left(1-\frac{C_{p}}{C_{0}}\right)*100\left[\%\right]$$

where *C_p_* is the concentration of the component in the permeate (mg L^−1^), and *C*_0_ is theconcentration of the component in the feed mixture (mg L^−1^) [18,19].

The fouling resistance (*Rf*), the total hydraulic resistance (*Rt*), and irreversible resistance (*Rirr*) were determined according to Darcy’s law and the resistance in-series model as follows:

(3) $$Rf=\frac{TMP}{\mu *Jp}-Rm\;\left[m^{-1}\right]$$

(4) $$Rirr=\frac{TMP}{\mu *Jpire}-Rm\;\left[m^{-1}\right]$$

(5) $$Rr=Rf-Rirr\;\left[m^{-1}\right]$$

(6) $$Rt=Rm+Rirr+Rr\;\left[m^{-1}\right]$$

where *µ* is the dynamic viscosity of permeate (0.00089 (Pa s) at 25 °C); Jpire is the flux of distilled water under filtration conditions after rinsing the membrane (L m^−2^ h^−1^). The relative value of the individual resistance was determined as the ratio of the reversible resistance, *Rr*, or irreversible resistance, *Rirr*, to the fouling resistance, *Rf*. 

## 3. Results and Discussion

The analysis of the results was divided into two parts to identify the mechanisms of combined organic–inorganic fouling. Firstly, the UF of the HA feed water and the influence of humic acid as a natural organic matter foulant on the flux, retention, and fouling resistances were discussed. Afterwards, the UF of organic–inorganic feed water and the influence of the addition of colloidal silica nanoparticles to the natural organic matter mixture were analyzed.

### 3.1. Analysis of Fouling during Humic Acid Feed Water

**Figure 2:**
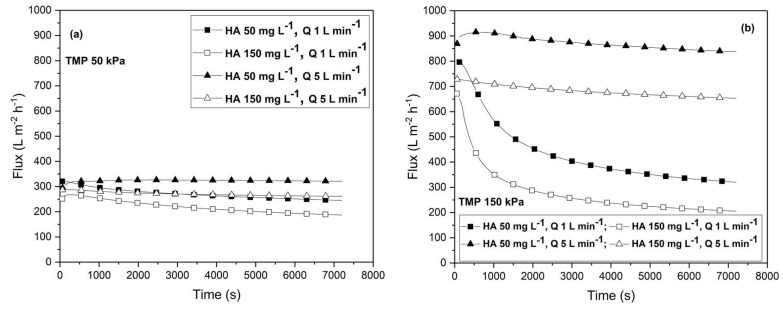
Time dependency of the permeate flux at the different operating conditions: (**a**) transmembrane pressure of 50 kPa and (**b**) transmembrane pressure of 150 kPa. The concentrations of humic acid (50 and 150 mg L^−1^) and the cross-flow rate (1, 5 L min^−1^) are varied.

Figure 2 shows the permeate fluxes obtained under the transmembrane pressure of 50 kPa (a) and 150 kPa (b) for various combinations of cross-flow rates (1 and 5 L min^−1^) and humic acid concentrations (50 and 150 mg L^−1^). As expected, the permeate flux decreased with increasing humic acid concentrations for all combinations of the Q and TMP values. On the other hand, an increase in TMP and Q values increased the permeate flux. The difference between the achieved permeate fluxes at higher cross-flow rates and the influence of the concentration of humic acid was especially pronounced under higher transmembrane pressure values. 

In Figure 2a, the slight initial decline in the permeate flux can be observed, followed by the rapid establishment of a steady state. This is for the lower TMP of 50 kPa, the cross-flow rate of 5 L min^−1^, and the concentration of HA of 150 mg L^−1^. A similar result was found for the concentrations of HA of 50 and 150 mg L^−1^ at the cross-flow rate of 1 L min^−1^. The shape of the flux decline in time is practically independent of the operating conditions, and a steady state is rapidly established in the first minutes of filtration. Further, under a transmembrane pressure of 50 kPa, at a cross-flow rate of 5 L min^−1^, and under a concentration of HA of 50 mg L^−1^, the steady-state permeate flux of 320 L m^−2^ h^−1^ was established at the very beginning of the filtration, indicating that in-pore fouling may occur.

In Figure 2b, a rapid decline in the permeate flux in the first ten minutes under the higher TMP of 150 kPa at the cross-flow rate of 1 L min^−1^ for both concentrations of HA can be noticed. This indicates that membrane fouling is combined and that after the initial mild concentration polarization effect, the flux declines slowly due to the further deposition of HA particles on the membrane surface and within the pores. Due to the low cross-flow rate, there was no significant removal of HA particles from the membrane surface and back transport by diffusion. This was especially the case under the higher concentration of HA due to the low diffusion coefficient of HA agglomerates. Under these conditions, a steady-state flux is achieved slowly. When increasing the cross-flow rate to 5 L min^−1^, the shape of the flux curve changes in the sense that the steady state is reached practically from the beginning of the filtration for both concentrations of humic acid. The high cross-flow rate prevents the deposition of particles and increases the back diffusion of particles, especially for the low concentration of HA. Also, the smaller particles of humic acid enter into the pores of the membrane due to the high pressure imposed and removed surface fouling. For the low HA concentration of 50 mg L^−1^, a slight increase in permeate flux can be observed followed by a decrease caused by the trade-off between back transport under a high cross-flow rate and the dragging force of the permeate toward the membrane due to the high TMP. However, the high cross-flow rate took out larger particles of humic acid, lowering surface fouling and resulting in a high permeate flux of 850 L m^−2^ h^−1^.

**Figure 3:**
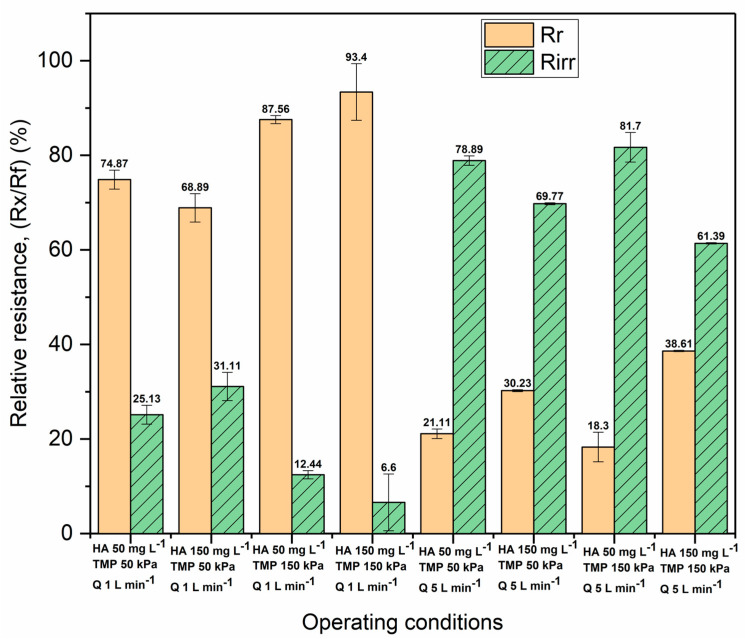
The relative values of irreversible (*Rirr*) and reversible resistance (*Rr*) during humic acid filtration.

Figure 3 shows the relative values of the irreversible (*Rirr*) and reversible resistances (*Rr*) calculated using Equations (3)–(5), while the absolute values are presented in the Supplementary Materials (Table S1 and Figure S5). The results show that humic acid causes both internal and surface fouling. Which one is going to be dominant depends on the operating conditions. Among others, the effect of the cross-flow rate is the key leading factor in dominantly irreversible fouling. Accordingly, at a cross-flow rate of 5 L min^−1^, irreversible fouling prevails compared to the effect of the cross-flow rate of 1 L min^−1^, where reversible fouling prevails. Further, the effect of the concentration of humic acid on the resistance can be observed. At the lower concentration of HA of 50 mg L^−1^, at the cross-flow rate of 5 L min^−1^, and at both values of TMP (50 and 150 kPa), internal fouling is more represented. Thus, at the cross-flow rate of 5 L min^−1^, the TMP of 150 kPa, and under the 50 mg L^−1^ HA concentration, the highest relative value of irreversible resistance of 81.7% was reached. On the other hand, at the cross-flow rate of 1 L min^−1^, under the TMP of 150 kPa, and under the HA concentration of 150 mg L^−1^, the highest relative value of reversible resistance of 93.4% was reached. Due to the agglomeration of HA, especially when the concentration is high and under low cross-flow rates, the membrane fouls mainly reversibly. Because of the establishment of a turbulent regime, under the rate of 5 L min^−1^, the agglomerates can be separated, but also, the particles are removed from the membrane surface by high shear stress. This prevents the formation of a cake layer, so HA macromolecules enter the pores of the membrane, causing predominantly irreversible fouling. 

Other scientists have also reported [4,20,21] the tendency of humic acid to agglomerate and deposit on the surface of the membrane alongside the adsorption and penetration of small particles into the pores. However, which mechanism will be dominant depends on the pores’ size. During the UF of HA in a stirred cell, it was found that adsorption and concentration polarization caused relatively little flux decline while HA aggregates had a significant effect on fouling only for the membranes with larger cut-offs [4]. Further, under lower concentrations of HA, the agglomeration is smaller, so the macromolecules can penetrate the pores of a membrane [4,22,23]. The presence of fouling, especially in-pore fouling, can affect separation efficiency in terms of reduced retention, so it is necessary to analyze retention as well. 

**Figure 4:**
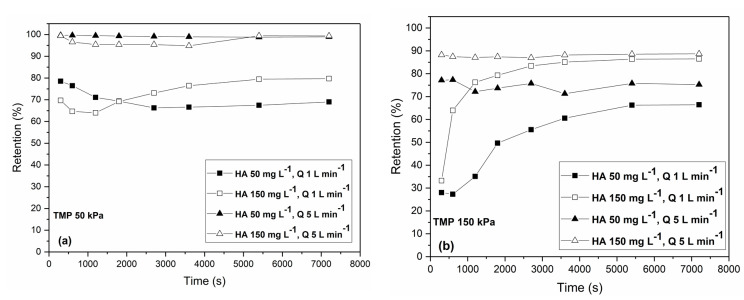
Time dependency of retention of humic acid at the different operating conditions: (**a**) transmembrane pressure of 50 kPa and (**b**) transmembrane pressure of 150 kPa. The concentrations of humic acid (50 and 150 mg L^−1^) and the cross-flow rates (1 and 5 L min^−1^) are varied.

Figure 4 shows the retention of humic acid by the membrane under the TMP values of 50 kPa (a) and 150 kPa (b) for various combinations of cross-flow rates (1 and 5 L min^−1^) and concentrations of HA (50 and 150 mg L^−1^). At the beginning of the filtration, retention is usually lower when a low cross-flow rate was imposed. Under the high cross-flow rate, retention is almost constant throughout all processes. Upon the achievement of steady-state conditions, retentions range from 60 to almost 100%, depending on the operating conditions. The highest retention of almost 100% was yielded under the highest cross-flow rate of 5 L min^−1^ and the lowest TMP, 50 kPa, regardless of the HA concentration (Figure 4a). On the other hand, with increases in the transmembrane pressure, retention declines (Figure 4b). At the higher TMP of 150 kPa, the higher retentions of about 85% were yielded for the higher concentration of HA of 150 mg L^−1^ for both the tested cross-flow rates. This is due to the greater agglomeration at the higher concentrations of HA. The lowest retentions of about 65% were obtained under the lowest concentrations of HA of 50 mg L^−1^ and the lowest cross-flow rate of 1 L min^−1^ under both values of TMP. The lower retention is achieved under the higher TMP and cross-flow rate and the lower concentration of HA due to the smaller agglomeration and the higher force imposed on the membrane by pressure. This indicates that a certain level of agglomeration and the formation of a dynamic cake layer at the membrane surface serving as the additional filtering layer can improve the retention of HA if operating conditions are adjusted properly. Even though in-pore fouling is predominant when the highest cross-flow rate is imposed, the retentions are high because of the intensive removal of the particles from the surface of the membrane. 

Lowe and Hossain [24] studied the cross-flow ultrafiltration of HA-containing feed water using polymeric membranes of 3, 5, and 10 kDa. Among the tested membranes, the 10 kDa membranes provided the highest permeate flux of about 60 L m^−2^ h^−1^ and the highest removal of HA under the low initial concentration of 15 mg L^−1^. They observed that the fouling was mainly reversible for all tested membranes. In our study, the 100 kDa membrane provided high fluxes while maintaining relatively high retention under the carefully chosen operating conditions. However, the performance of the membrane process can change when various constituents are present in feed water due to their interactions. 

### 3.2. Analysis of Fouling during Filtration of Organic–Inorganic Feed Water 

**Figure 5:**
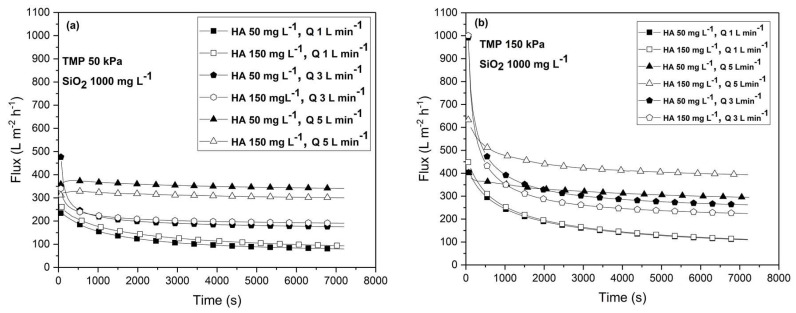
Time dependency of permeate flux under the different operating conditions: (**a**) transmembrane pressure of 50 kPa and (**b**) transmembrane pressure of 150 kPa. The concentrations of humic acid (50 and 150 mg L^−1^) and the cross-flow rates (1, 3, and 5 L min^−1^) are varied. The concentration of colloidal silica was constant (1000 mg L^−1^).

Figure 5 shows the time dependency of flux during the UF of a complex organic–inorganic mixture under the TMP of 50 kPa (a) and 150 kPa (b), under the various combinations of cross-flow rate (1, 3, and 5 L min^−1^), and under the concentrations of HA (50 and 150 mg L^−1^) for the colloidal silica concentration of 1000 mg L^−1^. The obtained fluxes are significantly lower for organic–inorganic feed water compared to HA feed water due to the presence of colloidal silica. Thus, the fouling is exacerbated by the addition of SiO_2_ particles. The exception is the operation under the cross-flow rate of 5 L min^−1^ and the low TMP of 50 KPa, where the fluxes are slightly higher for the organic–inorganic feed compared to HA feed. Under the cross-flow rate of 5 L min^−1^, the TMP of 50 kPa, and the HA concentration of 50 mg L^−1^, the highest permeate flux value of 340 L m^−2^ h^−1^ is obtained. The reason for this behavior is that the surface fouling caused by colloidal silica leads to the precipitation of HA agglomerates on the fouling layer, thereby preventing the entering of macromolecules of HA into the pores under the low TMP of 50 kPa.

Generally, the flux increased with increasing cross-flow rates for both the tested transmembrane pressures. The influence of humic acid concentration was almost negligible under the lower TMP (Figure 5a) and under the lowest cross-flow rate under the higher TMP (Figure 5b). Only when the high cross-flow rate of 5 L min^−1^ was imposed, a slight difference in fluxes between the 50 and 150 mg L^−1^ HA concentrations could be observed for the TMP of 50 kPa. Under the higher value of TMP of 150 kPa (Figure 5b), the effect of the HA concentration increased with the increasing the cross-flow rate. The influence of the HA concentration was negligible under the lowest cross-flow rate of 1 L min^−1^ since the same fluxes were obtained for both concentrations. However, unusual behavior occurred when the high cross-flow rate of 5 L min^−1^ was imposed. Namely, the flux was higher under the 150 mg L^−1^ concentration of HA than for the 50 mg L^−1^ concentration of HA and amounted to 394 L m^−2^ h^−1^. This indicates that the presence of SiO_2_ can change the nature of membrane fouling by HA under high cross-flow rates and transmembrane pressures. 

In Figure 5a, the trends of flux decline with time during filtration at the cross-flow rates of 1 and 3 L min^−1^ were the same with a rapid initial decline as a consequence of concentration polarization, followed by a gradual decline until the achievement of a steady state. The gradual decline of flux is the result of the deposition of colloidal silica particles, and of the creation of a cake layer on which humic acid particles precipitate especially at a lower cross-flow rate and transmembrane pressure. On the other hand, at the cross-flow rate of 5 L min^−1^, under the TMP of 50 kPa, and under both concentrations of humic acid, the initial flux decline was not observed. A very slight flux incline followed by a decline can be observed at the very beginning of filtration. Furthermore, the steady-state flux is established rapidly. This is because the high cross-flow rate of 5 L min^−1^ prevented the initial deposition of particles on the membrane’s surface. 

In Figure 5b, under the higher TMP of 150 kPa, the trend of flux decline with time is the same for all cross-flow rates with the rapid initial decline of the flux due to the concentration polarization followed by the slow deposition of particles until the achievement of a steady-state flux. The exception is the filtration of the 50 mg L^−1^ HA feed under the cross-flow rate of 5 L min^−1^, where the initial flux decline was not so expressed. Under other combinations of the concentrations of HA and SiO_2_ and the operating conditions, this was not observed (see Supplementary Materials, Figure S1b). 

**Figure 6:**
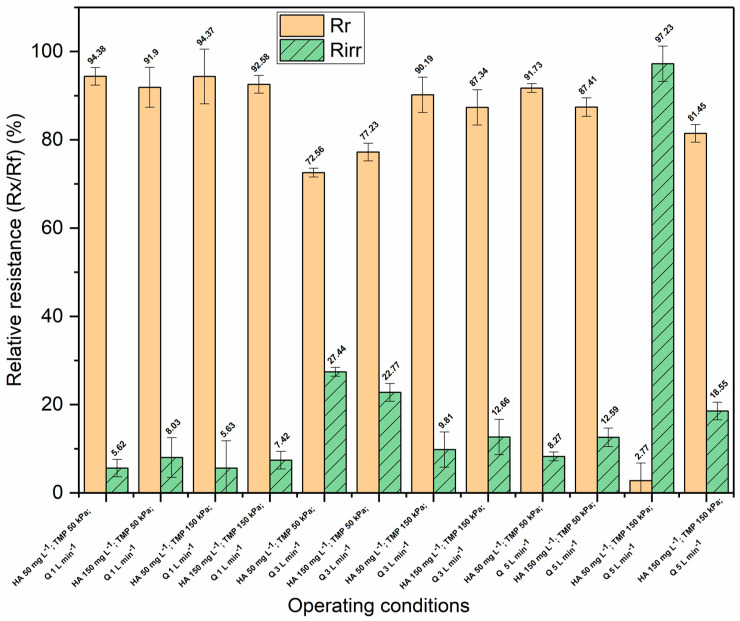
The relative values of irreversible (*Rirr*) and reversible resistance (*Rr*) during the ultrafiltration of a complex mixture of humic acid and colloidal silica.

Figure 6 shows the values of the irreversible (*Rirr*) and reversible resistances (*Rr*) relative to the overall fouling resistance for the organic–inorganic feed water, while the absolute values are presented in the Supplementary Materials (Table S2 and Figure S6). It can be noticed that during the filtration of the organic–inorganic mixture, reversible fouling was dominant under all the operating conditions except for the HA concentration of 50 mg L^−1^ under the TMP of 150 kPa and the cross-flow rate of 5 L min^−1^. Predominantly reversible fouling indicates that colloidal silica causes reversible fouling in the form of a cake layer and partially prevents the penetration of HA macromolecules into the membrane pores. This is particularly the case under low cross-flow rates and the high transmembrane pressures. At the cross-flow rates of 1 and 3 L min^−1^, the reversible resistances had higher absolute values, in the range of 40.3 × 10^10^ to 430 × 10^10^, than the irreversible resistances did, which were in the range of 13.1 × 10^10^ to 34.4 × 10^10^. Also, at a cross-flow rate of 5 L min^−1^, the reversible resistances had higher values, except under a transmembrane pressure of 150 kPa and a humic acid concentration of 50 mg L^−1^, where a significantly higher absolute value of irreversible resistance (148 × 10^10^) was obtained compared to the value of reversible resistance (4.21 × 10^10^) (Supplementary Materials, Table S2 and Figure S6). Reversible fouling was predominant under the concentration of 100 mg L^−1^ of HA and under the concentration of 500 mg L^−1^ of SiO_2_, and it was the lowest under the cross-flow rate of 5 L min^−1^ (Supplementary Materials, Figure S3). 

Given that HA agglomerates more at high concentrations and that the colloidal silica cake layer is compressible, predominantly reversible fouling can be expected even under a high cross-flow rate. On the other hand, when the concentration of HA is low under a high cross-flow rate, agglomeration is negligible or absent due to high turbulence. When, additionally, a high TMP is imposed, HA macromolecules can penetrate the SiO_2_ cake layer into the pores, so irreversible fouling becomes dominant. A high transmembrane pressure leads to the cake layer’s compression generally, and when a lower cross-flow is imposed, fouling remains predominantly reversible, while irreversible fouling is significantly reduced regardless of the HA concentration. 

**Figure 7:**
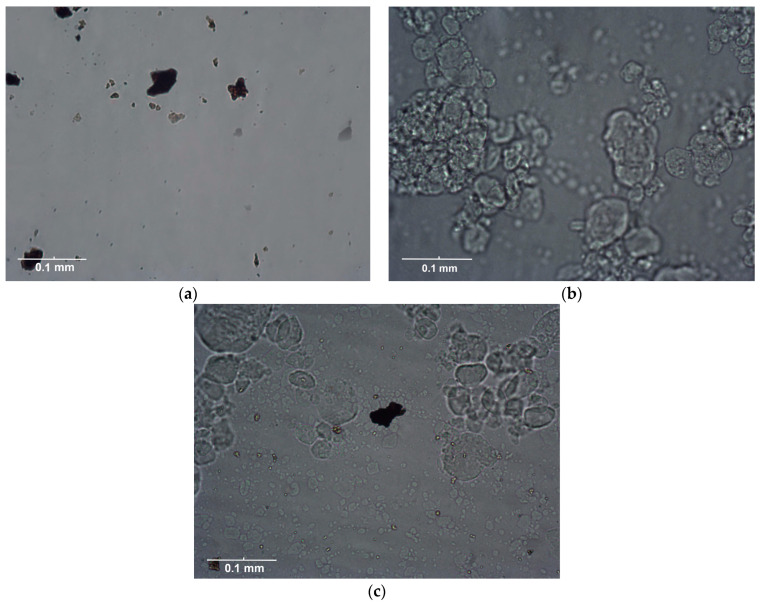
Microscopic images of the solution of (**a**) a humic acid concentration of 150 mg L^−1^, (**b**) a colloidal silica concentration of 1000 mg L^−1^, and (**c**) a mixture of humic acid and colloidal silica before filtration.

**Figure 8:**
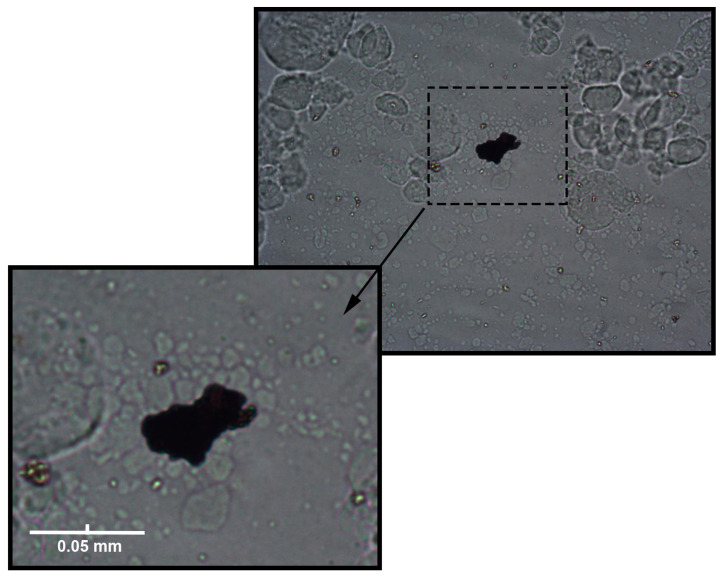
Magnified view of agglomerated and precipitated humic acid on the colloidal layer achieved under 10× *g* magnification of a light microscope.

For the confirmation of the observations made in our study, microscopic images of the feed waters were taken and are shown in Figure 7. Figure 7a shows a microscopic image of the feed water containing a HA concentration of 150 mg L^−1^. Figure 7b shows a microscopic image of the feed water containing the colloidal silica at a concentration of 1000 mg L^−1^. Figure 7c shows the feed water containing HA and silica in concentrations of 150 and 1000 mg L^−1^, respectively. For the feed water containing HA and SiO_2_, the deposited agglomerated HA on the colloidal silica layer could be observed. Figure 8 shows the magnified view of agglomerated and precipitated humic acid on the colloidal layer. Similar evidence of agglomeration and the deposition of humic acid, obtained by a transmission electron microscope, was given by Li et al. [25]. Also, similar observations were reported by Qin et al. [13]. They observed that the cake layer was created by the separate deposition of colloidal silica and humic acid onto the membrane surface during membrane distillation. Additionally, the fouling layer morphology showed that the humic acid was layered on top of the colloidal silica. 

Several researchers discussed the tendencies of HA and colloidal SiO_2_ to compress [4,22,23,25]. Humic acid has a low compressibility and causes internal pore fouling, while colloidal silica is highly compressible and forms a dynamic surface layer. Also, at a lower concentration of HA (50 mg L^−1^), lower agglomeration of humic acid occurs, which leads to the incorporation of macromolecules of HA into the pores.

**Figure 9:**
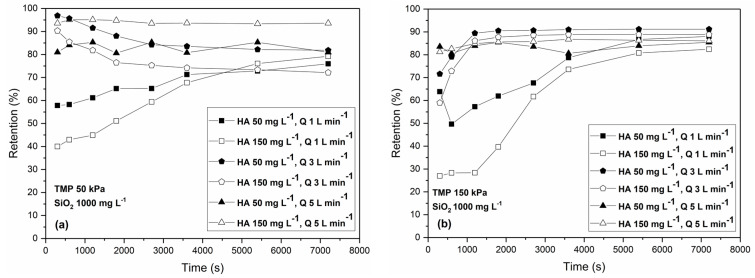
Time dependency of retention of humic acid during the ultrafiltration of the mixture of humic acid and colloidal silica under different operating conditions: (**a**) transmembrane pressure of 50 kPa and (**b**) transmembrane pressure of 150 kPa. The concentrations of humic acid (50 and 150 mg L^−1^) and cross-flow rates (1, 3, and 5 L min^−1^) are varied. The concentration of colloidal silica was constant (1000 mg L^−1^).

Figure 9 shows the retention of humic acid, during the filtration of the organic-inorganic mixture, under the TMP values of 50 kPa (a) and 150 kPa (b), for all combinations of Q (1,3, and 5 L min^−1^) and HA concentrations (50 and 150 mg L^−1^) and at a constant concentration of colloidal silica—1000 mg L^−1^. The retentions of HA during the filtration of the organic–inorganic mixture were generally higher compared to the retentions of HA during the filtration of the feed water containing HA only. Under the high cross-flow rate of 5 L min^−1^, the retention was practically constant during filtration. This was due to the lower fouling generally. For the low cross-flow rate, the retention was low at the beginning of the filtration and increased with time but did not reach the values obtained under the high cross-flow rates. Under these conditions, fouling was intensive, and it took time for the dynamic filter cake to form. During the filtration under the cross-flow rate of 3 L min^−1^, a decrease in retention occurred under the TMP of 50 kPa. 

The highest retention of almost 95% was yielded when the concentration of HA was 150 mg L^−1^, the cross-flow rate was 5 L min^−1^, and the low TMP was 50 kPa (Figure 9a). This is a slightly lower value than that of the filtration of the HA feed water despite the presence of a surface fouling layer at the membrane surface. Under low TMP values, the surface fouling layer was not compressed enough, so the small HA particles could penetrate it into the pores. Under the high TMP value of 150 kPa, the retentions ranged from 80 to 90%, the highest retentions obtained under the cross-flow rate of 3 L min^−1^ (Figure 9b). Under these conditions, the dynamic cake layer formed due to the not-so-high shear stress under the higher cross-flow rates of 5 L min^−1^, while the high TMP caused the compression of the cake layer and simultaneously pushed the small HA particles into the pores. The retention results additionally confirm the complexness of the influence of operating conditions on the agglomeration of HA and the formation and compressibility of a colloidal cake layer. Also, the presence of colloidal silica nanoparticles can increase the retention of HA by properly adjusting the operating conditions. In other studies where multi-walled carbon nanotubes and graphene oxide particles were used and incorporated into the PES membrane, the HA rejection values were 90.8% and 94.8%, respectively [26]. Also, Almanassra et al. [27] used carbon derived from carbide oxidized by acid treatment (OCDC) as a filler in the production of innovative PES composite membranes. The results demonstrate a significant advancement in HA rejection rates, with a range between 92.6 to 96.8%. 

Previous studies on humic acid and mixtures of humic acid and colloidal silica have primarily focused on polymer membranes. To compare the effectiveness of ceramic and polymer membranes at removing humic acid, we have provided a comparative table in this study (Table 1). The removal rate of humic acid is influenced by various factors, including the type and pore size of the membrane. Our ceramic membrane demonstrated a high capacity for removing humic acid, both as a single foulant and in combination with colloidal silica, indicating its effectiveness for water treatment applications.

## 4. Conclusions

In this study, an analysis of the fouling of a ceramic membrane by complex organic–inorganic feed water containing high concentrations of humic acid and colloidal silica was presented. The colloidal SiO_2_ nanoparticles in the organic–inorganic feed water exacerbated the fouling of the membrane, yielding lower fluxes (109–394 L m^−2^ h^−1^) compared to the HA feed water (205–850 L m^−2^ h^−1^). On the other hand, the retentions obtained under the presence of SiO_2_ were higher except for the highest cross-flow rates. Under the high cross-flow rates, such as 5 L min^−1^, the agglomeration of HA was the least and the dynamic fouling layer was disrupted, so the smallest HA particles could penetrate the pores. Unlike during the filtration of HA where irreversible resistance prevailed under a highly turbulent regime, during the filtration of the organic–inorganic mixture, reversible resistance appeared under all the operating conditions, except in one case. The exception is the filtration of the organic–inorganic mixture of a 50 mg L^−1^ HA concentration, which resulted in a lower flux than the one of 150 mg L^−1^ HA concentration under the TMP of 150 kPa and the cross-flow rate of 5 L min^−1^. Here, the irreversible fouling was unexpectedly overcome. This was due to the specific interaction between the humic acid and colloidal silica, under a certain combination of concentrations of both constituents, transmembrane pressure, and cross-flow rate. By the proper operation under mild operating conditions, i.e., lower TMPs and moderate cross-flow rates, fouling can be mitigated, and relatively high fluxes can be yielded with high retentions even in the presence of nanoparticles.

## Figures and Tables

**Table 1:** Review of investigated studies on humic acid removal.

Membrane Type	Pore Size/MWCO	Type of Foulants	Rejection of HA (%)	References
Ceramic ZrO_2_ with α-alumina support	100 kDa	HA	65–99	This work
		HA-SiO_2_	75–95	
Polymeric UF RC	30 kDa	HA-SiO_2_	55	[4]
Polymeric PES	30, 50, 100, 300 kDa, 0.16 µm	Soil-based HA	50–99	[5]
Polymeric PC	0.2 µm	HA	>80	[20]
Polymeric PES	3, 5 and 10 kDa	HA	70–80	[24]
PES/MWCNTs PES/GO	59 kDa	HA	91–95	[26]
PES/CDC	34.9–36.5 nm	HA	92.6–96.8	[27]
Polymeric PCTE	100, 300 kDa	Soil-based HA and SRHA	87–95	[28]
Polymeric PES	1 M			
Polymeric PVDF	0.16 µm			

## Data Availability

The data presented in this study are available on request from the corresponding author.

## Supplementary Materials

The following supporting information can be downloaded at: https://www.mdpi.com/article/10.3390/membranes13030334/s1.
