# Supplementary material for: New Insights into the Fouling of a Membrane during the Ultrafiltration of Complex Organic–Inorganic Feed Water
Source: Membranes (Basel). 2023 Mar 14;13(3):334. doi: 10.3390/membranes13030334 (PMC10054249; doi:10.3390/membranes13030334)
Supplement: Supplementary file 1 [file membranes-13-00334-s001.zip › membranes-2225370-supplementary.pdf]

membranes

Supplementary Material

New Insights into the Fouling of a Membrane during the Ultrafiltration of Complex Organic–Inorganic Feed Water

Vedrana Prorok [1], Dejan Movrin [2], Nataša Lukić [1,*] and Svetlana Popović [1,*]

[1] Faculty of Technology Novi Sad, University of Novi Sad, Bulevar cara Lazara 1, 21000 Novi Sad, Serbia
[2] Faculty of Technical Sciences Novi Sad, University of Novi Sad, Trg Dositeja Obradovića 6, 21000 Novi Sad, Serbia
* Correspondence: nlukic@tf.uns.ac.rs (N.L.); svetlana.popovic@uns.ac.rs (S.P.)

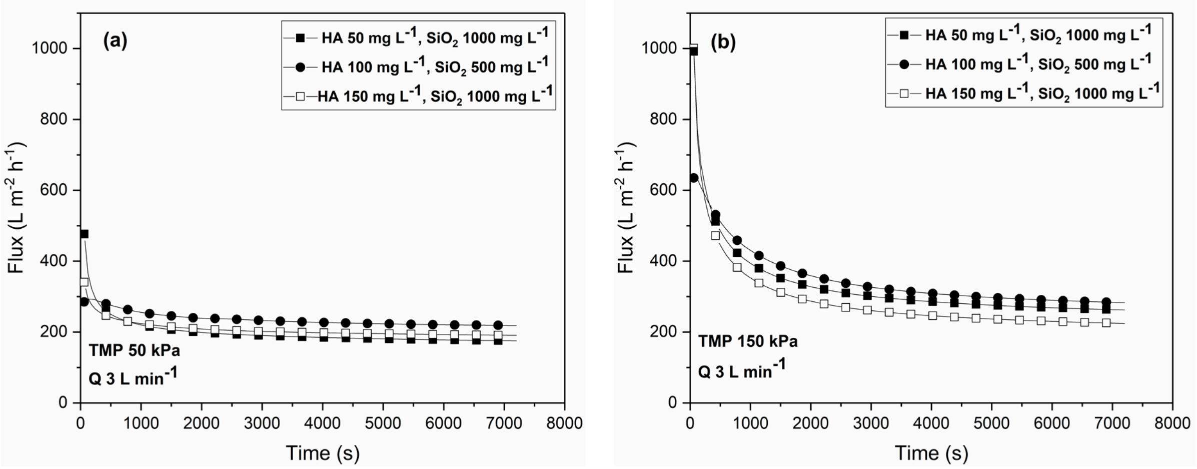

(**a**) (**b**)

Figure S1. Time dependency of permeate flux at (a) transmembrane pressure of 50 kPa and (b) transmembrane pressure of 150 kPa. The concentration of humic acid (50, 100, 150 mgL^{-1}) and colloidal silica (500, 1000 mgL^{-1}) are varied. The cross-flow rate of 3 $Lmin^{-1}$ was constant.

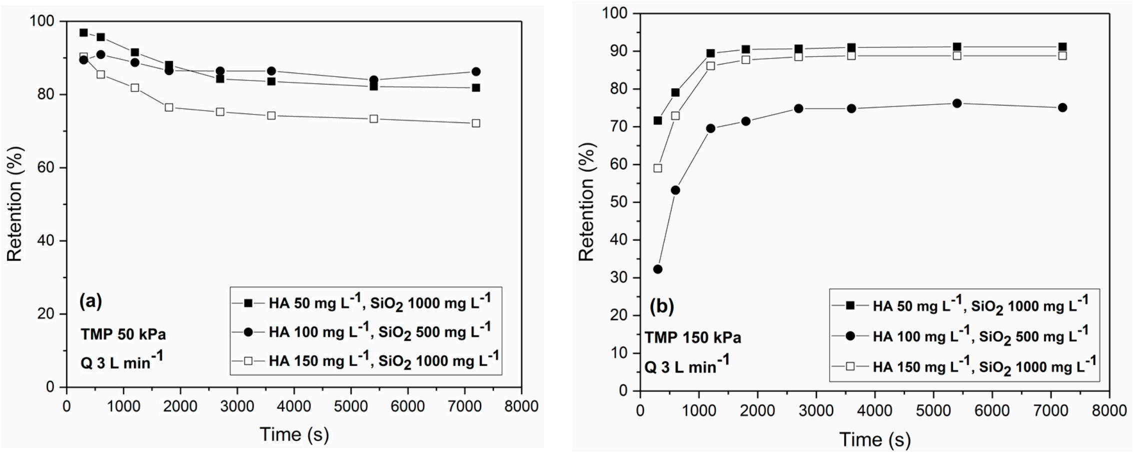

(**a**) (**b**)

Figure S2. Time dependency of retention of humic acid at (a) transmembrane pressure of 50 kPa and (b) transmembrane pressure of 150 kPa. The concentration of humic acid (50, 100, 150 mgL^{-1}) and colloidal silica (500, 1000 mgL^{-1}) are varied. The cross-flow rate of 3 $Lmin^{-1}$ was constant.

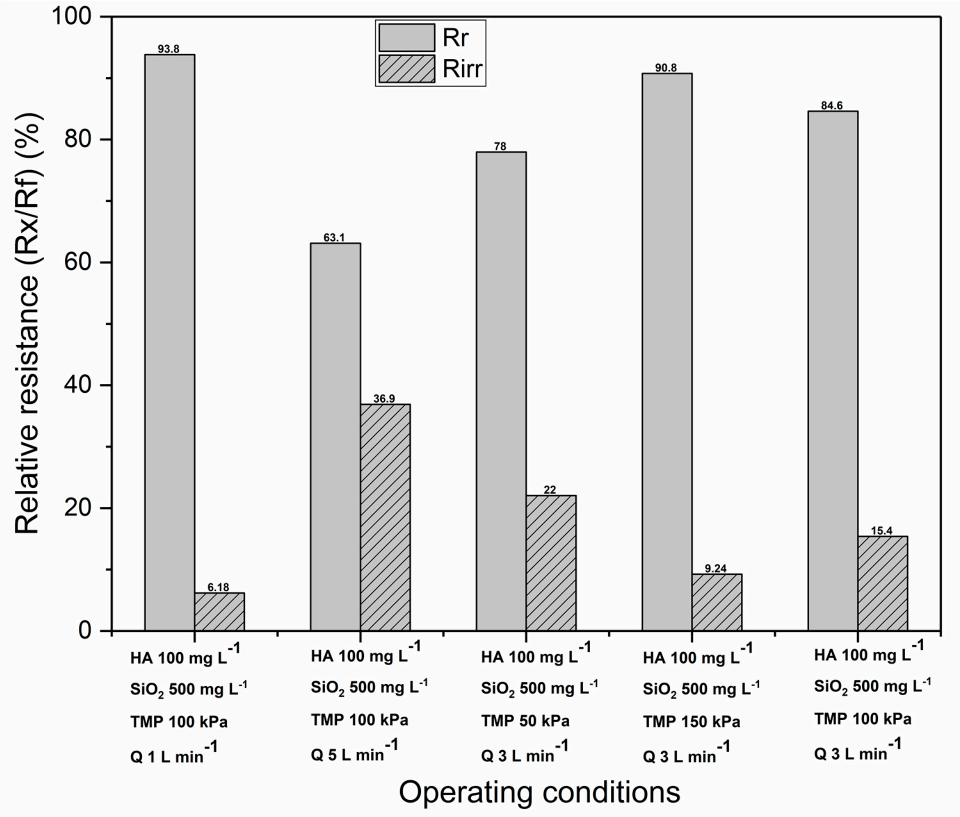

Figure S3. The relative values of irreversible (Rirr) and reversible resistance (Rr) during the ultrafiltration of a complex mixture of humic acid with colloidal silica.

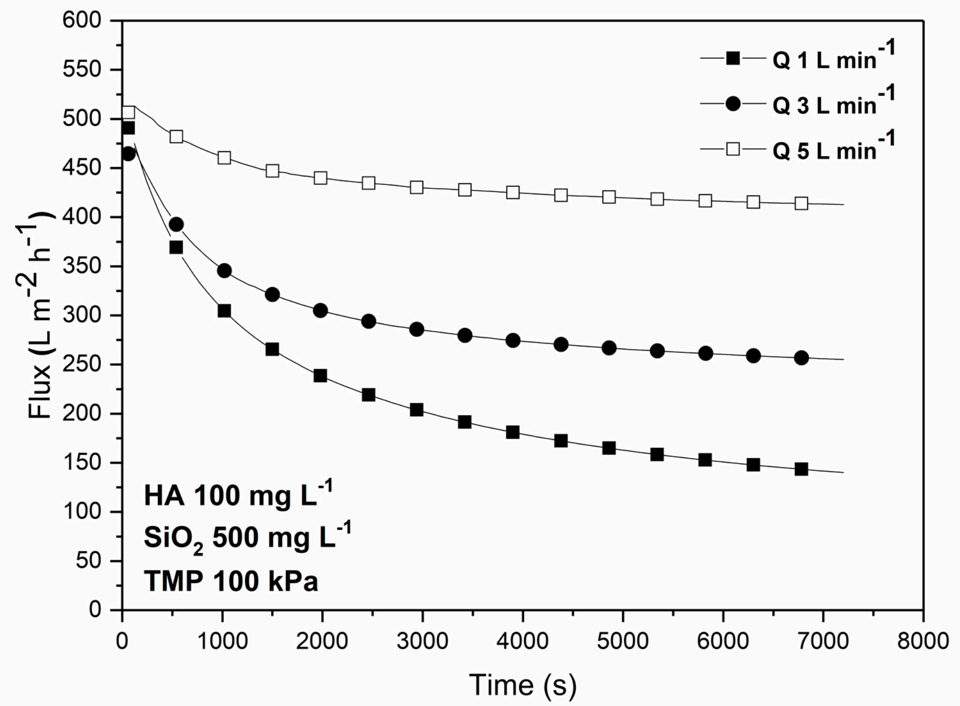

Figure S4. Time dependency of permeate flux during ultrafiltration of the mixture of humic acid and colloidal silica at the different cross-flow rates.

Table S1. Absolute values of reversible (Rr) and irreversible resistance (Rirr) at humic acid filtration.

Operating conditions	Mean absolute values of Rr resistance with ± STDEV (× 10^{10} m^{-1})	Mean absolute values of Rirr resistance with ± STDEV (× 10^{10} m^{-1})
HA 50 mg L^{-1}; TMP 50 kPa Q 1 L min^{-1}	21.4 ± 1	7.18 ± 0.01
HA 150 mg L^{-1}; TMP 50 kPa Q 1 L min^{-1}	38 ± 0.67	19.5 ± 2.66
HA 50 mg L^{-1}; TMP 150 kPa Q 1 L min^{-1}	114 ± 5.98	15.7 ± 1.14
HA 150 mg L^{-1}; TMP 150 kPa Q 1 L min^{-1}	220 ± 17.7	17.3 ± 1.34
HA 50 mg L^{-1}; TMP 50 kPa Q 5 L min^{-1}	1.93 ± 0.01	7.16 ± 0.01
HA 150 mg L^{-1}; TMP 50 kPa Q 5 L min^{-1}	8.13 ± 2.13	17.7 ± 1.68
HA 50 mg L^{-1}; TMP 150 kPa Q 5 L min^{-1}	3.24 ± 1.43	14.6 ± 2.11
HA 150 mg L^{-1}; TMP 150 kPa Q 5 L min^{-1}	14.7 ± 0.44	23.9 ± 0.04

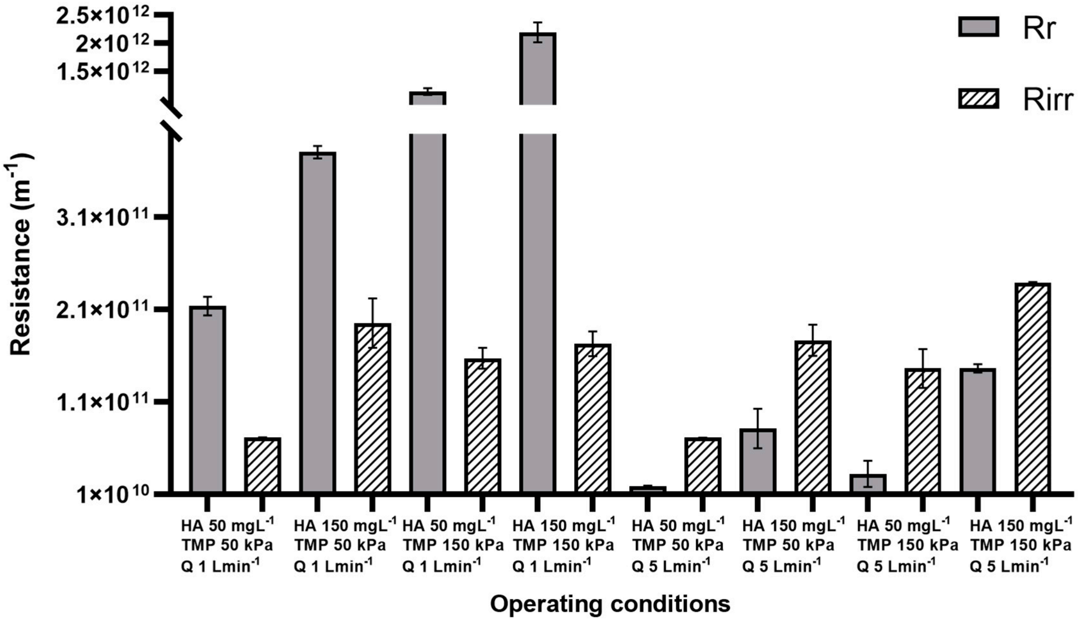

Figure S5. Absolute values of reversible (Rr) and irreversible resistance (Rirr) at humic acid filtration.

Table S2. Absolute values of reversible (Rr) and irreversible resistance (Rirr) during the ultrafiltration of a complex mixture of humic acid with colloidal silica.

Operating conditions	Mean absolute values of Rr resistance with ± STDEV ($\times 10^{10}$ m^{-1})	Mean absolute values of Rirr resistance with ± STDEV ($\times 10^{10}$ m^{-1})
HA 50 mg L^{-1}; SiO_2 1000 mg L^{-1}; TMP 50 kPa; Q 1 L min^{-1}	179 ± 9.71	16.7 ± 5.45
HA 150 mg L^{-1}; SiO_2 1000 mg L^{-1}; TMP 50 kPa; Q 1 L min^{-1}	150 ± 19.6	13.1 ± 3.
HA 50 mg L^{-1}; SiO_2 1000 mg L^{-1}; TMP 150 kPa; Q 1 L min^{-1}	401 ± 70.6	26.1 ± 2.05
HA 150 mg L^{-1}; SiO_2 1000 mg L^{-1}; TMP 150 kPa; Q 1 L min^{-1}	430 ± 20	34.4 ± 4
HA 50 mg L^{-1}; SiO_2 1000 mg L^{-1}; TMP 50 kPa; Q 3 L min^{-1}	44.7 ± 4.03	16.9 ± 1.91
HA 150 mg L^{-1}; SiO_2 1000 mg L^{-1}; TMP 50 kPa; Q 3 L min^{-1}	40.3 ± 1.6	11.9 ± 0.8
HA 50 mg L^{-1}; SiO_2 1000 mg L^{-1}; TMP 150 kPa; Q 3 L min^{-1}	160 ± 39.9	17.4 ± 2
HA 150 mg L^{-1}; SiO_2 1000 mg L^{-1}; TMP 150 kPa; Q 3 L min^{-1}	189 ± 32.1	27.4 ± 2
HA 50 mg L^{-1}; SiO_2 1000 mg L^{-1}; TMP 50 kPa; Q 5 L min^{-1}	5.27 ± 0.31	1.99 ± 1.54
HA 150 mg L^{-1}; SiO_2 1000 mg L^{-1}; TMP 50 kPa; Q 5 L min^{-1}	10.6 ± 0.73	1.53 ± 0.07
HA 50 mg L^{-1}; SiO_2 1000 mg L^{-1}; TMP 150 kPa; Q 5 L min^{-1}	4.21± 0.09	148 ± 28.5
HA 150 mg L^{-1}; SiO_2 1000 mg L^{-1}; TMP 150 kPa; Q 5 L min^{-1}	81.5 ± 1.09	18.6 ± 3.83

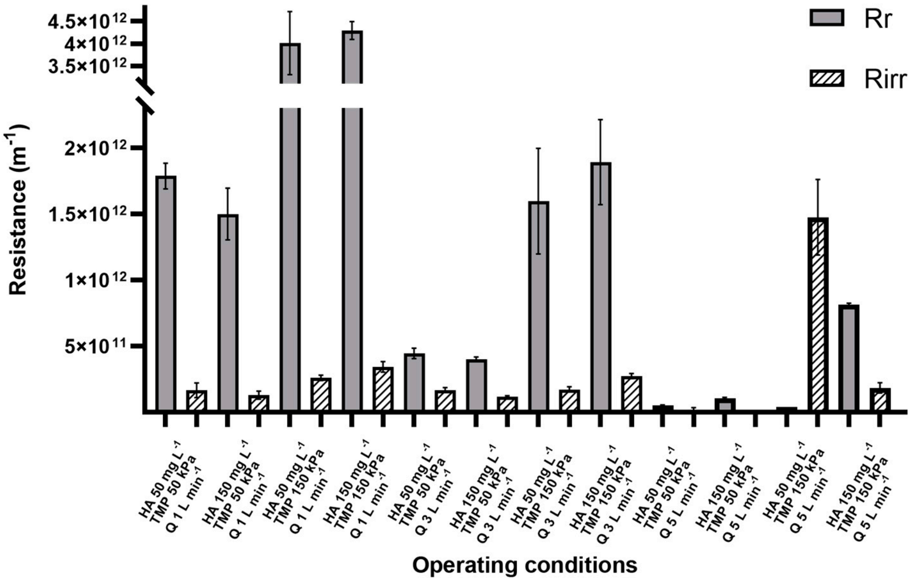

Figure S6. Absolute values of reversible (Rr) and irreversible resistance (Rirr) during the ultrafiltration of a complex mixture of humic acid with colloidal silica.
